# Shared decision making in Eosinophilic esophagitis: Integrating physician and patient perspectives

**DOI:** 10.1371/journal.pone.0350662

**Published:** 2026-06-10

**Authors:** Kerry A. Ryan, Kelcie K. Darpel, Joel H. Rubenstein, Evan S. Dellon, Lauren P. Wallner, Raymond De Vries, Joy W. Chang

**Affiliations:** 1 Center for History, Humanities, Arts, Social Sciences and Ethics in Medicine (CHHASSEM), University of Michigan Medical School, Ann Arbor, Michigan, United States of America; 2 Division of Gastroenterology, Department of Internal Medicine, University of Michigan, Ann Arbor, Michigan, United States of America; 3 Veterans Affairs Center for Clinical Management Research, Ann Arbor VA Medical Center, Ann Arbor, Michigan, United States of America; 4 Division of Gastroenterology and Hepatology, Center for Esophageal and Swallowing Disorders, University of North Carolina School of Medicine, Chapel Hill, North Carolina, United States of America; 5 Division of General Medicine, Department of Internal Medicine, University of Michigan, Ann Arbor, Michigan, United States of America; University of Connecticut, UNITED STATES OF AMERICA

## Abstract

**Background and objectives:**

Shared decision making (SDM) is a patient-centered approach for conditions where multiple, preference-sensitive treatment options exist and there is no single best choice. Eosinophilic esophagitis (EoE), an increasingly prevalent chronic immune-mediated disease, offers an example of how patients and physicians use SDM to navigate the challenging tradeoffs of weighing pharmacologic and dietary therapies. We aimed to identify communication challenges for SDM in EoE care from physician and patient perspectives and to explore how patient treatment preference archetypes influence SDM.

**Research Design and methods:**

We conducted a qualitative study with one-on-one, semi-structured interviews with adult patients with EoE and physicians. Patients (n = 35) were recruited from a single academic center (mean age 41 years [(SD 15.2]; 51% male; predominantly White 83%). Physicians included gastroenterologists (n = 9) and allergists (n = 7) from varied practices across Michigan. Interview guides were informed by the Theoretical Domains Framework and iteratively refined. Interview transcripts were coded using both deductive and inductive strategies and analyzed thematically via descriptive content analysis.

**Results:**

Three central themes emerged: 1) Patients often lack knowledge or have misconceptions about EoE and its treatments, but differed in how and from where they want to gain disease-focused information, 2) Physicians generally supported SDM, but patients varied in how they want to be involved in decisions about their care, and 3) Both patients and physicians wanted accurate and user-friendly informational resources about EoE to support effective communication and SDM.

**Conclusions:**

Our analysis revealed that patients with EoE have diverse preferences for disease-related learning, decision making, and communication. There is a clear need for accurate, accessible, and personalized information to improve patient-physician understanding and communication, without which SDM and patient-centered care in EoE cannot be achieved. What we learned can be applied to other health conditions where there is clinical equipoise between various effective management options.

## Introduction

Shared decision making (SDM) is a patient-centered approach to managing conditions where multiple, preference-sensitive treatment options exist, none of which are clearly favored by evidence.[1] Eosinophilic esophagitis (EoE), a chronic immune-mediated disease with increasing prevalence, offers a lens for understanding how patients and physicians navigate SDM in the context of complex tradeoffs between pharmacologic and dietary therapies in a situation of clinical equipoise.[2–4] EoE is characterized by eosinophilic inflammation and fibrosis of the esophagus, causing significant symptom burden – including dysphagia, food impaction, reflux and regurgitation, and chest pain – in both children and adults, and is associated with reduced health-related quality of life, increased healthcare use, and high healthcare costs.[5–7] EoE can be effectively treated with several therapies, including elimination diets and medications – including proton pump inhibitors (PPI), topical corticosteroids, and novel biologics – each with distinct tradeoffs in efficacy, side effects, and lifestyle impact.[8,9] As comparative efficacy data is lacking and no single therapy is clearly superior in all patients, clinical guidelines recommend a SDM approach.[4] This approach requires: 1) clear, evidence-based knowledge about options to weigh benefits, risks, and uncertainties; 2) effective, tailored communication with patients; and 3) support in clarifying patient values and preferences.[10] This shared approach promotes patient autonomy by involving them in treatment plans, resulting in greater patient buy-in and adherence, and potentially improved health outcomes.[11–14]

Despite growing awareness and guideline endorsement of SDM in electing EoE treatments, nearly half of EoE patients report little or no involvement in care decisions.[15] To deliver patient-centered, high-quality EoE care, we must understand the barriers to SDM. Without insight into how patients and physicians communicate about EoE and share information during clinical encounters, meaningful SDM will not be realized.

There is a growing but still limited body of literature on SDM in EoE, leaving health care professionals without the information they need to navigate complex decision and communication challenges with patients.[16,17] In our prior work, we identified three distinct treatment preference archetypes among EoE patients – medication preference, natural treatment preference, and treatment ambivalence – a finding that underscores the need for personalized management of the disease instead of a “one size fits all” approach.[18] In this qualitative study, we build on our prior quantitative work by integrating both physician and patient perspectives and applying patient treatment preference archetypes to qualitative interview data to explore the best ways to deliver knowledge, overcome problems in physician-patient communication, and identify desired resources to support effective shared decision making.

## Materials and methods

### Physician interviews

To better understand physician experiences, attitudes, and preferences for the management of EoE, we conducted 45-minute semi-structured Zoom interviews with gastroenterologists and allergists across Michigan. Informed by the Theoretical Domains Framework, we developed and iteratively refined an interview guide with input from a multidisciplinary group of experts in EoE, decision sciences, medical sociology, and qualitative methods.[19] During interviews, physicians were asked to think back on recent encounters with patients with EoE to describe: 1) their communication about EoE with patients; 2) perceived patient knowledge about EoE including the sources of that knowledge; 3) physician treatment strategies and preferences related to EoE; 4) perceived patient preferences and values related to EoE; 5) physician experiences in managing EoE cases, including challenges they faced; and 6) their own and perceived patient definitions of ‘treatment success’ related to EoE (see Supporting Information, **S1 File**: Physician Interview Guide).

We purposively sampled gastroenterologists and allergists to account for age, gender, race/ethnicity, and practice setting (Table 1). Physicians were recruited by email invitation through the Michigan Allergy & Asthma Society and the Michigan Gastrointestinal Society listservs, and a Michigan Medicine physician contact list from October 25, 2022 to February 2, 2023. Upon completion of the interview, participants were sent a $50 check or gift card as compensation for their time. All interviews were Zoom recorded, transcribed verbatim, and de-identified for analysis. This study was exempt from federal regulations by the University of Michigan IRB (HUM00170564).

**Table 1:** Physician participant demographics.

	Physicians, n (%)
**Gender**	
Female	8 (50%)
Male	8 (50%)
**Age, Mean (SD)**	45.8 (9.1)
**Race** White Asian or Asian American Middle Eastern/North African	10 (62.5%) 5 (31.2%) 1 (6.3%)
**Ethnicity**	
Hispanic/Latine	0 (0%)
Physician Type Allergist Gastroenterologist	7 (43.8%) 9 (56.3%)
**Years in Practice** Less than 5 5–10 11-20 20+	3 (18.8%) 5 (31.3%) 6 (37.5%) 2 (12.5%)
**Primary Practice Setting** Academic medical center/university-based Community health system (non-academic) Community private practice Veterans affairs/federal facility	8 (50.0%) 4 (25.0%) 3 (18.8%) 1 (6.2%)

### Patient interviews

To understand patient perspectives on managing EoE and identify factors influencing treatment decisions, we conducted one-hour interviews with patients as part of their participation in a Q-methodology study, via Zoom videoconferencing.[18] This method combines qualitative and quantitative approaches to systematically study subjective viewpoints.[20] Adult patient participants (≥18 years) with a confirmed diagnosis of EoE were recruited from the University of Michigan’s gastroenterology and allergy clinics (Table 2) from March 16, 2023 to June 12, 2023. Participants used QMethod software (qmethodsoftware.com) to rank their values and preferences related to EoE.[21] During Q-method interviews, patients were asked about the following domains as they related to EoE: goals and priorities, disease perception and burden, treatment preferences, treatment burden and risk, information seeking, and communication preferences with physicians (see Supporting Information, **S2 File**: Patient Interview Guide). Archetypes were assigned based on how each patient’s Q-sort loaded onto factors derived from by-person factor analysis; participants whose Q-sorts were highly correlated were grouped into the same archetype, representing a discrete viewpoint on EoE management. Of the 35 participants, 17 loaded onto the medication preference archetype, 7 onto the natural treatment preference archetype, 6 onto the treatment ambivalent archetype, and 5 did not load onto any archetype. A detailed description of our EoE Q-method study, methods, and quantitative findings are previously published.[18] Both sets of interviews were conducted by trained qualitative researchers with expertise in psychology and medical sociology (KD, KR).This study was deemed exempt from federal regulations by the University of Michigan IRB (HUM00170559).

**Table 2:** Patient participant demographics.

	Patients, n (%)
**Gender**	
Female	17 (48.6%)
Male	18 (51.4%)
**Age, Mean (SD)**	40.9 (15.2)
**Race**	
White Asian or Asian American Black or African American Native Hawaiian or Pacific Islander Other/Multi-race	29 (82.9%) 1 (2.9%) 2 (5.7%) 1 (2.9%) 2 (5.7%)
**Ethnicity**	
Hispanic/Latine	4 (11.4%)

### Data analysis

Initial coding schemes for each set of interviews were developed both deductively and inductively, based on interview guide domains and review of transcripts by the study team. For physician interviews, the coding scheme was iteratively refined through in-depth readings and discussions of a subset of six transcripts. Three team members (JC, KD, KR) then applied the coding scheme to one gastroenterologist and one allergist interview to further refine the scheme, and the finalized coding scheme was applied to all remaining transcripts. For patient interviews, the coding scheme was similarly developed and then refined through initial coding of four interviews—representing each of the three patient archetypes identified through Q-method analysis, and one additional interview that did not align with any archetype.[18] All transcripts were double-coded using MAXQDA 2022 and 2024 qualitative software, with discrepancies resolved by consensus to ensure consistency and accuracy in analysis.[22,23] Thematic saturation was assessed iteratively during analysis and no new themes emerged after reviewing the final transcripts in both the physician and patient datasets. Finally, to enhance the rigor, MAXQDA AI Assist tools were used post-hoc, after all human coding and preliminary analysis were complete, to generate summaries and query via prompts within specific coded segments, such as “What are the main takeaways from these coded segments?” and “What are the best quotes representing this theme?” to serve as a post-hoc cross-check for completeness and representativeness. Final interpretations, verification, and analyses were conducted by the study team, which included physicians, qualitative methodologists, and social scientists, who maintain full responsibility for all coding and analytic decisions. Physician quotes are labeled with ‘G’ for gastroenterologists (G01–G09) and ‘A’ for allergists (A01–A07), reflecting specialty and sequential interview order; patient quotes use ‘P’ followed by a three-digit de-identified code (e.g., P113). Additional illustrative quotes are provided in Supporting Information, S1 Table: Supplementary Quotes.

## Results

### Physician perspectives

Gastroenterologists (G) and allergists (A) in our interviews described a range of challenges encountered when educating patients about EoE, as well as strategies they used with patients to support SDM. In the following sections, we present key themes related to: (1) challenges related to patient knowledge gaps; (2) approaches to patient-physician communication and decision making; and (3) physician use of information resources and their unmet needs for educational materials.

### Challenges related to patient knowledge gaps

When caring for EoE patients, physicians reported several challenges related to patient knowledge, including unfamiliarity with the diagnosis and reliance on internet sources of information. Physicians perceived significant gaps between patient understanding and the realities of EoE and its management, which negatively impacted shared decision making. Misconceptions about potential complications, disease chronicity, and unrealistic expectations about outcomes posed significant challenges. As one gastroenterologist noted, “I feel like most of my patients generally don’t know much about EoE. I feel like it’s a new diagnosis. It’s a new terminology.” (G04) Similarly, an allergist pointed out that “the majority of my patients don’t even know the name of their diagnosis.” (A04) One physician made the distinction between awareness and understanding: “[Patients] may know they have EoE, but they have absolutely no idea what EoE is.” (A03) As another specialist noted, “[Patients] don’t have a great concept of […] the chronicity, or the fact that it’s like asthma. It flares and it doesn’t flare, but it’s very chronic.” (A05) However, some physicians noted that levels of patient knowledge about EoE can vary widely. One remarked, “I think it really differs per patient. I’ve had some patients—specific to this disease—come in after their diagnosis. When they have been seeking additional sources of information like Facebook support groups, those are on the end of the more informed.” (A04)

Physicians expressed concern about the comprehensibility and accuracy of sources of information gleaned from googling, social media, or other sources, which can add to the complexity of the visit. For example, one gastroenterologist noted: “That’s kind of the pattern I see with most patients, is they will look up a little bit, but most of them are—because it can get confusing […] essentially, looking up stuff on the internet becomes very confusing.” (G05) An allergist pointed out, “not all the information on the internet is accurate. There was some education that needed to be done as well.” (A01)

According to physicians in our study, patients commonly misunderstood treatment options, which also complicated shared decision making. Particularly, confusion around dietary therapy sometimes resulted in unrealistic expectations specifically regarding the implications of implementing an elimination diet, how to identify and avoid potential food triggers, and how long to maintain dietary changes. Discussing allergy testing for EoE, one physician commented: “The perception it seems from patients [is] that they get tested, the test is positive, they eliminate that food and they never have any symptom ever again and they don’t do a full elimination.” (A04) Another stressed the importance of getting into specifics when it comes to diet therapy: “Because if you don’t get into specifics, then yes, they say, ‘Oh, well, I want to do the diet. Sounds great. All I have to do is change my diet,’ without understanding the fact that they’re signing themselves up for multiple endoscopies, days off of work, and things like that.” (G06) Similarly, physicians had to field concerns about medications. One described misconceptions regarding PPIs: “There’s a lot of stuff like that’s out on the internet about the dangers of PPIs and things, some founded and some which haven’t been proven yet.” (G05) Others discussed unrealistic expectations about medication use: “Sometimes people want the quick cure. ‘Wait, if I take these steroids, will it be gone forever?’” (G06)

### Physician views on effective communication and decision making

Despite these patient knowledge barriers, physicians in our study acknowledged that EoE treatment can be preference-sensitive and recognized the importance of effective patient-physician communication and a shared decision making approach, which considers patient concerns, values, and preferences. With multiple effective treatment options, one physician stated: “Because in my mind, all three classes of treatment options are equally viable. I think it’s more important to choose a treatment that’s in line with the patient’s preferences rather than being overly rigid.” (G03) Another remarked: “I’m just a big believer in autonomy […] don’t live your life, and I don’t walk in your shoes and if that’s your choice, that’s your choice […] I think I always try to just make sure that they know what treatments are available.” (A04)

Some physicians noted that this shared approach can promote patient autonomy, buy-in, and adherence, and ultimately, could yield better outcomes.[11] As one allergist stated: “I usually offer all of them, because I usually like to do kind of a shared decision making with the patient, because I feel like they’ll buy into the treatment more if they get to make part of the decision.” (A02) A gastroenterologist emphasized the connection between choice and adherence: “If you provide them a choice, they’re more likely to adhere than if I just tell them what they’re going to do.” (G04)

Despite these intentions, some specialists reflected on cases where these efforts fell short, leading to suboptimal outcomes. Physicians identified communication gaps as one barrier. One admitted: “Maybe, I didn’t take the time to assess how well they actually understand what I’m saying. Sometimes you just tell them, and you think you did your job, but do they actually understand or hear the importance of it?” (G01) Another discussed challenges with establishing trust:

I think that sometimes patients—you don’t have their trust. Or you haven’t established their trust, or when they’re not responding, for instance, to the first medication that you put them on, and they lose trust in that relationship or don’t want to continue for that reason. (G06)

### Physician information use and resource needs

Physicians in our study used a wide variety of resources to educate patients about EoE. Some used pamphlets and handouts to offer basic information on symptoms and treatments. Physicians also directed patients to websites (e.g., the American Partnership for Eosinophilic Disorders, the Mayo Clinic) for additional information. Some used stock patient education “smart phrases” in the electronic medical record, visual aids, or drew diagrams of the esophagus to help patients better understand. Occasionally, information about EoE and its management was included with biopsy result letters sent to patients [24,25].

However, physicians noted several unmet educational resource needs. Both gastroenterologists and allergists wanted standardized, comprehensive, and accessible education materials and websites that cover treatment options and risks, as well as current advancements in EoE, presented in patient-friendly language. One physician noted that existing websites lack detailed information specific to EoE and are difficult to navigate: “I think the biggest thing is […] a better website would be better, that’s for the patients.” (A03)

Given these unmet education needs, physicians expressed interest in comprehensive educational tools that could present the risks, benefits, and lifestyle implications of each option in a clear and accessible format. One gastroenterologist specifically suggested that decision aids could fill this gap:

This would be the right place to put a decision aid or something along those lines. If I had a go-to website […] or a piece of paper for which I say, “This is your EoE, and here’s your options. Which one does it sound the best to you, which I’m sure exists?” That would probably be helpful for patients, just in general. (G04)

Such tools could also help assess patient preferences, values, and concerns regarding different treatment options: “Figuring that sort of thing out like how do you feel about medications in general? How do you feel about—if we recommended avoiding foods, how realistic is that for you? That kind of information I think would be helpful.” (A06)

### Patient perspectives

Mirroring physicians’ viewpoints, patients’ attitudes and values played a significant role in making decisions about EoE. In the quantitative analysis of our Q-method study, we previously identified three preference archetypes around EoE treatments: 1) medication preference driven by symptoms and desire to minimize risk of complications, 2) natural treatment preference focused on identifying trigger foods and diet adherence, and 3) treatment ambivalent viewing EoE as mild and episodic with low treatment priority.[18] Analysis of our interview data revealed that patient (P) attitudes toward gaining information and knowledge, communication with physicians, and making decisions, varied based on these different archetypes.

### Information and knowledge seeking

Patients in all archetypes identified themselves as ‘knowledge seeking’. However, the extent of their knowledge seeking and what ‘knowledge seeking’ meant differed across archetypes. These varying definitions of ‘knowledge seeking’ highlight the importance of tailoring communication and the decision making process to accommodate individual patients’ information needs and preferences.

#### Medication preference archetype.

For the medication preference archetype, most participants relied on their physician for information and did not actively seek EoE-related knowledge from other sources (e.g., searching the internet, using social media, talking to family or friends). Many of these participants expressed concerns about the quality of online information. As one participant explained: “I don’t go to like Dr. Google because I know that that’s not a good source of information. I like it from the medical professionals, the doctors, especially the GI doctors.” (P113) Another pointed out: “…anything that I read or see, until I take stock in it, I pretty much verify with my doctor. I don’t want to go down the black hole of misinformation.” (P119) These participants also discussed that since other people’s experiences and bodies differ, confirming information with a physician is important. As one participant observed: “I feel like someone’s circumstances can be different than my own, so it might not be as relevant. […] I just listen to what the doctor thinks should be done.” (P108)

#### Natural treatment archetype.

Among the three archetypes, the natural treatment archetype tended to be the most overtly knowledge seeking. They were the most likely to proactively search for evidence-based information online, often taking the lead in researching EoE and its treatments. One participant stated: “I did a lot of research on the computer -- a lot of YouTube videos. I listened to a lot of professors speak at universities, and I tried to go with credible sources.” (P110) This archetype also emphasized the importance of being their own advocates and engaging with and learning from health care professionals:

Ask questions and make sure that you have a provider that’s going to answer those questions. If you don’t, just be pushy about it. I hate that we have to be all our own advocates, but you need to be your own advocate in your own healthcare journey. Be pushy. Ask the questions. (P132)

#### Treatment ambivalent archetype.

Compared to the natural treatment archetype and similar to the medication preference archetype, patients who were treatment ambivalent were less proactive in their knowledge seeking and tended to rely on healthcare professionals for information: “One of my doctors had sent me some resources. I’m sure the link is in my portal profile, but I haven’t really looked into anything EoE related for a while.” (P130) This archetype also tended to gather information about EoE from social media sources. As one explained: “I go to YouTube and hear other people’s stories or I go to the discussion groups on Facebook….” (P109)

### Decision making and communication

Across archetypes, patients shared some commonalities in their attitudes toward decision making and communication. Patients wanted clear communication and to be involved in treatment decisions; however, they varied in their preferred approaches and their reliance on medical expertise.

#### Medication archetype.

The medication archetype generally preferred physician-led decision making, because they trusted their doctors, valued medical expertise, and were inclined to follow their instructions, given the view that patients have less medical expertise. As one participant explained: “The doctor is educated on this, and I am not, so I’ll just follow their opinions. I’m not a doctor, so I should not be making medical decisions without consulting one. […] I just listen to what the doctor thinks should be done.” (P108) Patients in this archetype valued having communication and explanations from their physician: “I don’t make any medical decisions without running through my doctor. […] I like my doctor’s input. I would never do anything without getting their input.” (P125)

#### Natural treatment archetype.

The natural treatment archetype tended to prefer a more balanced approach with both the patient and the physician providing input. These patients pointed to the importance of being their own advocates, and some specifically mentioned wanting to make their own decisions with physician input. As one participant explained it: “I actually like to make a lot of decisions about my EoE on my own, but I do take input from the doctor. I do advocate for myself.” (P110)

This archetype also valued having an open dialogue with their physician, where the physician was knowledgeable about options, but also listened to the patient to better understand their preferences and values. One patient found it “very irritating and very frustrating” when physicians took a paternalistic approach: “I like when doctors communicate with me and talk through me like I’m a person. […] I just want it to be more of an open dialogue instead of one way…” (P132)

#### Treatment ambivalent archetype.

This archetype also tended to prefer a shared decision making approach. These patients valued the physician’s input but were not willing to just follow the doctor’s lead without first having a dialogue. As one patient commented, “I would rather that we have a conversation with doctors. The doctors tell me about the research, tell me about what works right now for most people. Then we make a decision together.” (P122) Another pointed out, “I think it’s important for anybody when they’re talking to a doctor to not just follow their lead blindly, to actually question them.” (P130) Finally, another stated: “The days of not asking the doctor what’s going on, just taking their advice is over.” (P128)

### Patient resource needs

Similar to physicians, patients, regardless of archetype, wanted educational and decision-making resources that were accessible, understandable, and came from trustworthy sources. They wanted a clear and comprehensive overview of EoE, including its causes, symptoms, impacts and risks, and available treatment options. One patient pointed out that they wanted “more accessible information from verifiable sources,” rather than relying on potentially unverified internet searches. (P108) Another would like to see “a less clinical description of EoE that is more layman’s terms.” (P126) Another patient wanted EoE treatment information “in clear language--all the options and why, pros and cons of each one, and why this one is being recommended,” and emphasized the importance of information tailored to their own circumstances, preferences, and values, “… the doctor discussing *my* case and *my* treatment options and my risks. That’s helpful, but the pamphlet ‘What’s EoE?’ that doesn’t help…” (P128)

Several patients also wanted the opportunity to learn from and connect with other patients with EoE: “Honestly, talking to somebody else who had it would’ve probably helped tremendously. Even if they’d only taken one avenue, I think that would help a lot.” (P119) Another wanted help finding support groups “because that is one other great piece of information to have, and to talk to other people so you don’t feel like you’re the only crazy one who can’t swallow” (P120)

There was no clear consensus on the best modality for patients to receive this information. Some patients wanted information in the form of a physical brochure or handout, while others expressed a preference for online or web-based information, perhaps with links to further information. And there were those who wanted to ensure good communication and dialogue with their physician, perhaps with visual aids.

## Discussion

This study explored perspectives of gastroenterologists, allergists, and patients on communication and decision making in EoE care. We found that both physicians and patients face significant challenges in SDM implementation. Physicians reported that patients often have knowledge gaps about EoE and rely on potentially inaccurate online information. They also identified gaps in available educational materials and expressed desire for standardized resources. Patients across all three treatment preference archetypes (medication preference, natural treatment preference, and treatment ambivalent) valued being knowledge seekers, but differed in their information-seeking behaviors, preferred communication styles, and decision-making approaches including reliance on their doctor’s opinions and management decisions. Both groups identified the need for improved, accessible educational materials to support effective SDM.

Because EoE is a newly defined disease with rapidly evolving treatments, the need for clear, accessible, and accurate information and decision support is acute. Several studies give a glimpse of physicians’ treatment choices, but to our knowledge, this is the first qualitative study to integrate both physician and patient perspectives and examine communication challenges through the lens of distinct patient treatment preference archetypes, providing deeper understanding of these challenges.

Our work adds to a growing literature showing unmet needs in clinical conversations about EoE. In a large cross-sectional survey of adult patients and caregivers of children with eosinophilic gastrointestinal diseases, Hiremath et al. found that many patients encountered health care professionals who lacked EoE knowledge, with over half requiring care transfers to find knowledgeable specialists.[26] In our prior studies, EoE patients described frustrating encounters with physicians lacking EoE expertise.[16] In a separate cohort of adults with EoE and caregivers of children with EoE, many reported a desire to seek physicians who were supportive of their treatment choices, and nearly half (42%) experienced little shared decision making in their EoE care.[15] Conversely in our present study, physicians’ views of patients’ unfamiliarity and misconceptions about EoE call attention to an increased burden on physicians to provide accurate and clear knowledge, and an opportunity ripe for a decision aid that will enable structured presentation of treatment options with their risks and benefits, tailored to individual patient values and preferences. While physicians may direct patients to online EoE-related websites, these resources vary considerably in depth, accuracy, and currency, further underscoring the unmet need for standardized, evidence-based educational materials for patients with EoE.

Our study responds to this need by identifying addressable barriers to SDM. Despite growing awareness and support for SDM in EoE, there remains limited guidance and resources to do so, leaving physicians to navigate complex treatment decisions and patient preferences alone.[4] By including a range of patients with EoE across all preference archetypes and physicians from diverse clinical disciplines and practice settings, we captured a broader understanding of communication dynamics and limitations of current EoE-related education. Additional strengths of our study include rigorous qualitative methods including team-based iterative coding and analysis and leveraging an innovative AI-Assist application to summarize human-coded segments.

However, this study has some limitations. As a qualitative study, these findings may not be generalizable to all physicians or EoE patients. As EoE management relies on specialist expertise, including dietary counseling, management of atopic disease, and endoscopy, our physician sample was limited to specialists (gastroenterologists and allergists), which excludes the perspectives of primary care providers (PCPs) and may not capture the full range of health care professional experiences with EoE management—especially considering that primary care often serves as the entry point for EoE patients. Inclusion of PCP perspectives would likely enrich our understanding of patient experiences, particularly regarding how patients first learn about EoE and communication challenges at earlier stages of care. The patient participants were drawn from a single academic center and may not reflect the experiences of patients in community settings or those with limited access to specialist care. Physician interviews were conducted shortly after FDA approval of dupilumab for EoE, and gastroenterologists may have had limited experience with this biologic compared to allergists already familiar with its use in atopic conditions; regardless, the expanding treatment landscape only underscores the importance of SDM as patients and physicians navigate increasingly complex treatment decisions together. While the overall sample size was modest, we achieved thematic saturation across both physician and patient interviews, suggesting that the core themes identified are robust and reflective of common experiences. Future research should also examine SDM and patient education needs in pediatric EoE populations, where parents and caregivers serve as primary decision-makers and present distinct communication challenges and priorities. Despite these limitations, our findings offer valuable insights into real-world challenges and opportunities to address patient-physician communication and bolster SDM in EoE care.

Our findings have important implications for EoE care and for SDM more broadly. Our prior work with this cohort suggests that diagnostic and treatment-specific concerns vary by archetype, with implications for treatment management and SDM.[18] This study expands on our earlier research by demonstrating that patient treatment preference archetypes also explain variation in how patients seek knowledge, handle information sources, and prefer to communicate with physicians. Each archetype seeks information from different sources (health care professionals, publications, internet, social media), calling for accurate and accessible resources in diverse educational formats. While all patients valued clear communication, they differed in preferred decision-making styles – from physician-led approaches to more collaborative partnerships. For physicians, recognizing these differences is critical for effective communication and SDM implementation, which may in turn support improved treatment adherence.[27,28]. If we are to embrace patient-centered care in EoE, patients must be equipped with evidence-based knowledge and physicians must tailor their communication and decision support strategies to individual preferences. The development of improved educational resources and decision support tools is essential, particularly given the rapidly evolving landscape of EoE treatments. These insights may also inform communication strategies and SDM approaches beyond EoE to other complex medical conditions where clinical equipoise exists, such as inflammatory bowel disease, asthma, and early-stage prostate cancer, which similarly require ongoing management and value-laden tradeoffs among benefits, risks, and treatment burden.

**Contributors:** All individuals who contributed to this study and manuscript met the criteria for authorship as outlined by the International Committee of Medical Journal Editors (ICMJE).

## Supporting information

S1 File Physician Interview Guide. (DOCX)

S2 File Patient Interview Guide. (DOCX)

S1 Table Supplementary Quotes. (DOCX)
