# Supplementary material for: Shared decision making in Eosinophilic esophagitis: Integrating physician and patient perspectives
Source: PLoS One. 2026 Jun 10;21(6):e0350662. doi: 10.1371/journal.pone.0350662 (PMC13252738; doi:10.1371/journal.pone.0350662)
Supplement: S1 File — (DOCX) [file pone.0350662.s001.docx]

**EOE PHYSICIAN INTERVIEW GUIDE**

We are conducting interviews to better understand provider experiences, attitudes, and preferences for the management of Eosinophilic esophagitis (EoE).

I’ll start about by asking some basic questions about your clinical work and experience treating EoE.

- How many years have you been in practice?
- Can you briefly tell me about your clinical work?
  - [For allergists only] What mix of adult vs. pediatric patients do you see?
- In the last month about how many patients have you seen with EoE?
  - [If none] how many patients have you seen in the past year?

Think back on one of your most recent new patients with EoE.

- Tell me about that patient encounter. What was that conversation like?
- What did the patient know about EoE walking in the door?
  - What do patients generally know about EOE when they walk in the door?
- How did you describe EoE to this patient?
  - If this patient asked you to explain what EoE is and what that means for them, what would you say?
  - What does EoE mean for them in the short-term?
  - What does EoE mean for them, if anything, in the long-term?
- What treatment or management strategies did you offer this patient?
- What alternatives did you offer this patient, if any?
  - [If no] Are there times that you offer alternatives to patients with EoE? What are those? (and why?)
  - [If yes] Do you usually offer these alternatives to patients?
  - Under what circumstances might you use different strategies?
  - Are there times that you *don't* offer multiple treatment options? Why or why not?
  - [If combo] Are there times that you *don't* offer a combination of these treatments? Why or why not?
- What recommendation, if any, did you make to the patient?
  - Is this your typical first choice for treating or managing patients with EoE? *Why or why not?*
- How did you figure out what the therapy to recommend to the patient?
  - What information did you use to make this recommendation?
- What information about the patient did you need to know to make this rec?
  - [If need clarification] clinical/objective information
- What information from the patient did you need to know to make this rec?
  - [If need clarification] preferences/values/subjective information
- Do you offer different treatment or management strategies to different types of patients? *Why or why not?*
- Did this patient talk about what they cared about most/matters most with regard their disease or its treatments?
  - What do patients with EoE care about most? What do you think patients prioritize when it comes to managing EoE?
  - What concerns do patients with EoE bring up most often? What tends to be their main concern?
  - What types of expectations or hopes do patients with EoE bring up most often? In general, what are their main hopes/expectations?
  - How is this different from what matters most to you, if at all?
- Tell me about a time when the management of an EoE case went well. What kind of conversations/discussions did you have with the patient? What aspects made the case go well?
  - What information did you + the patient discuss?
- Tell me about a time when the management of an EoE case went poorly/didn’t go well. What kind of conversations/discussions did you have with that patient? What aspects made the case go poorly?
- What are some of the challenges you have experienced when making treatment or health management recommendations to patients with EoE?
  - How do you typically handle [these challenges]?
  - Tell me about a conversation you had with a patient who wanted to pursue a different treatment from the one you recommended. How did you respond?
  - Tell me about a conversation you had with a patient who did not want to start a treatment right away. How did you respond?
- What does “treatment success” look like to you? What does “treatment success” look like to patients? Why do you think these two definitions of success do, or do not, align.
- Where do patients generally get their information? What do you think about those sources of information?
- What kinds of information/training/resources do you need, if anything, to have more productive conversations about EoE treatment options with patients?
- Finally, is there anything you’d like to add that we haven’t had a chance to talk about?
