# Supplementary material for: Shared decision making in Eosinophilic esophagitis: Integrating physician and patient perspectives
Source: PLoS One. 2026 Jun 10;21(6):e0350662. doi: 10.1371/journal.pone.0350662 (PMC13252738; doi:10.1371/journal.pone.0350662)
Supplement: S2 File — (DOCX) [file pone.0350662.s002.docx]

**EOE PATIENT Q-SORT FOLLOW-UP INTERVIEW GUIDE**

Now, I want to ask you about your experiences and preferences related to managing EoE, as well as your responses to the card sort activity.

1. First, could you tell me a bit about the time you were **first** diagnosed with EoE? When was it? How did the diagnosis come about?
   - What treatment or management strategies were offered?
   - How was it decided which treatment or management strategy to use?
   - Who was part of making that decision?
     - What clinicians?
     - Family or friends?
2. Have you ever changed or stopped treatment or management strategy for EoE? If so, what brought about that change?
3. Now, let’s turn to the card sorting activity. Thinking about the statements, what jumps out to you?
   - Did anything surprise about how you ranked the cards?
   - Were there any statements that were hard for you decide about? Why?
4. Look at the items you ranked as “most agree.” Why did you select these statements as “most agree”?
5. Look at the items you ranked as “most disagree.” Why did you select these statements as “most disagree”?
6. Was there anything missing from the card sort? Anything you think is important to consider in making decisions about EoE that was not included?
7. Setting the cards aside, what’s most important to you when managing/treating your EoE? And why?
8. What does “success” or a “good outcome” in EoE mean to you?
9. If you were talking to someone else with EoE, what would be your advice to them about choosing a treatment?
10. Where do you get information about EoE and EoE treatment or management strategies?
    - What resources or information do you get from friends or family, the internet, or social media, if any?
    - What do you think about these sources of information?
11. Is there anything that would have helped make the decision-making experience around EoE treatment easier or better for you?
    - Anything the doctors could have done?
    - Anything that healthcare system could have done?
    - Any materials or information that would have been helpful?

Finally, is there anything you’d like to add that we haven’t had a chance to talk about?
