# Supplementary material for: Shared decision making in Eosinophilic esophagitis: Integrating physician and patient perspectives
Source: PLoS One. 2026 Jun 10;21(6):e0350662. doi: 10.1371/journal.pone.0350662 (PMC13252738; doi:10.1371/journal.pone.0350662)
Supplement: S1 Table — (DOCX) [file pone.0350662.s003.docx]

**Supplementary Table 1. Supplementary quotes physician and patient interviews**

| **Section** | **Theme** | **Definition** | **Exemplary Quotes** |
| --- | --- | --- | --- |
| **PHYSICIAN PERSPECTIVES** |  |  |  |
|  | **Challenges Related to Patient Knowledge Gaps** | Physicians perceived significant gaps in patient understanding of EoE, including unfamiliarity with the diagnosis, disease chronicity, and treatment options, often compounded by reliance on inaccurate internet sources. | *She had a fairly decent sense of what the disease entailed, but again, not all the information on the internet is accurate. There was some education that needed to be done as well. (A01)* |
|  |  |  | *Some patients google their symptoms online and try to diagnose themself with it. Really, it depends on the patient, depends on the age of the patient, the education of the patients, how much they know, how much they do or don’t wanna know. Really, there's no rhyme or reason these days. A lot of patients know a fair amount about their diseases. Some of them don’t really know anything. (A01)* |
|  |  |  | *The problem is, any monkey that can code, can make a blog or a website. That's where you start getting into EoE is caused by vaccines. That type of information can make, on an individual level, care difficult for a patient. Just in general, can make the profession itself exhausting and draining over time. (A01)* |
|  |  |  | *You know, doctors are busy, and I understand that, but it does seem like a lot of the time the person that has diagnosed their EOE has not actually explained what it is to them. They come to me very confused. I would say I spend the majority of my initial visit with them counseling them on what EOE is and talking about the next steps. I would say most of them, their knowledge of what EOE is, is pretty limited when I first see them, especially if they're—I notice that especially if they're coming from outside of our GI clinic, maybe from an outside clinic, they just aren't really sure what's going on, and they weren't necessarily told the next steps in their management. (A02)* |
|  |  |  | *I'm thinking of several patients I've had where I've had to explain everything to them, where really they don't understand what EOE is, why they're taking an acid blocker medication for EOE, or, you know, what kind of treatments are even available. I do feel like I spend the majority of the visit counseling them on things that maybe should have been explained when they were diagnosed, but were not. (A02)* |
|  |  |  | *They just know that they have EOE and that's the reason why they're having their symptoms. […] They didn't really understand why or what exactly it is. (A03)* |
|  |  |  | *[Patients] may know they have EoE, but they have absolutely no idea what EoE is. Unfortunately, when they come from GI, they don't get a very good explanation of what really is going on, so I try to explain to them what the disease is, how it presents, some of the pathophysiology, and then what options are out there for treatment, what is the potential long-term ramifications of the disease. (A03)* |
|  |  |  | *The majority of my patients don't even know the name of their diagnosis. (A04)* |
|  |  |  | *It feels like I’m explaining something, and it doesn’t make sense at all to them. Then I struggle with figuring out what can I change about the way I’m presenting this so that I can give them that information. […] it feels like I hit a wall in terms of what I know and can communicate. (A04)* |
|  |  |  | *I think it really differs per patient. I've had some patients—specific to this disease—come in after their diagnosis. When they have been seeking additional sources of information like Facebook support groups, those are on the end of the more informed. (A04)* |
|  |  |  | *[Patients] don't have a great concept of […] the chronicity, or the fact that it's like asthma. It flares and it doesn't flare, but it's very chronic, and it's very hard to just like all of a sudden be okay without it. (A05)* |
|  |  |  | *They're also unclear on how to manage it. They assume that as long as they're not having symptoms after a dilation they don't really have to do anything, but the inflammatory cascade goes on… (A05)* |
|  |  |  | *[Patients] often think it's like a general food allergy. I usually tell 'em that it's not those type, you don't need an EpiPen, things like that. (A06)* |
|  |  |  | *If they’re coming in with symptoms and we say, ‘We think this is what’s going on,’ they know nothing about it. Some people have read about it in different places. Actually, our population in is pretty educated, and they actually, have usually picked some pretty good sources, but not 100 percent. The knowledge is not great walking in. I don’t think people know much about it.... (A07)* |
|  |  |  | *It is a whole, new world for them that they don’t understand. (A07)* |
|  |  |  | *I think the general public may not be as well aware of what we're seeing as far as our field was quite concerned about the rate of EoE over the last 20 years. […] I don't think the news is getting out there regarding EoE, the understanding of patients is still quite minimal… (G01)* |
|  |  |  | *This patient knew that she had a food impaction, had a, basically, a blockage of the esophagus. That was the extent of what she knew. (G03)* |
|  |  |  | *I feel like most of my patients generally don't know much about EoE. I feel like it's a new diagnosis. It's a new terminology. It’s not something that they’re very familiar with when I first make the diagnosis, whether it’s a food impaction that I saw in the ER or making it ’cause they came in my office with intermittent dysphagia or whatnot. It’s not something I feel like they’ve heard of quite a bit, so I do think it’s a relatively—some other conditions, IBS, Crohn’s, any celiac disease, they have these—they already heard about it. They’ve read about it. EoE is not one of those diseases where I feel like they come in with lot of knowledge. (G04)* |
|  |  |  | *That's kind of the pattern I see with most patients, is they will look up a little bit, but most of them are—because it can get confusing […] essentially, looking up stuff on the internet becomes very confusing. (G05)* |
|  |  |  | *[EoE information on the internet] I think that it's a little less consumer-friendly, let's say, and more so all you're finding is medical articles. Then don't have multiple websites that are telling them stuff. It's just this medical thing, so then they kind of give up. (G05)* |
|  |  |  | *Patients typically don't know very much at all. I think the way I usually explain it to the patients is that you have essentially inflammation, inflammatory cells in your esophagus that are causing your symptoms. It gets a little hairy when you're trying to talk to them about treatments because you're mentioning medications like PPIs. Then they go, Oh, it's acid reflux. It becomes challenging at times to explain the difference. Most patients really don't—beyond that, don't know a whole lot about the condition. (G06)* |
|  |  |  | *[Patient] came to me with at least a little bit of knowledge. Honestly, when you dig a little bit deeper, and you start talking about pathophysiology, explaining the importance of managing over time, you realize that that knowledge is fairly shallow in terms of really understanding what they're dealing with. Sometimes, patients will understand that there is some kind of dietary management. Again, often that's based on reading off of the Internet and things like that.(G06)* |
|  |  |  | *Because if you don't get into specifics, then yes, they say, 'Oh, well, I want to do the diet. Sounds great. All I have to do is change my diet,' without understanding the fact that they're signing themselves up for multiple endoscopies, days off of work, and things like that. (G06)* |
|  |  |  | *Sometimes people want the quick cure. 'Wait, if I take these steroids, will it be gone forever?' (G06)* |
|  |  |  | *I even sometimes have to catch myself and say this is an outpatient procedure. Because sometimes patients don't realize that. I think not assuming that people know what you're talking about is always a good policy when you're in clinic. Just starting basic, and then if they want more information, you can provide it, or if they already know it, then that's fine. No harm, no foul. It's better to start with the basics and build up. (G06)* |
|  |  |  | *Most people don't know much about it even if they have the entity, and that's typical of a lot of our patients. (G09)* |
|  | **Physician Views on Effective Communication and Decision Making** | Physicians recognized EoE treatment as preference-sensitive and valued shared decision making that incorporates patient concerns, values, and preferences, while acknowledging that communication gaps and trust barriers can undermine these efforts. | *I present all options to the patients and then let them participate in some type of shared decision-making process where they can give their input and feedback in terms to what could potentially work for them. […] You go over the risks, the benefits of alternatives of all treatment options with the patients. Does he or she feel comfortable? Do they have an understanding? Do they know what a response would be, a non-response would be, what the next step would be? Just wanna make sure that they have an understanding and a buy-in on their treatments because, I feel that if they do, you get away from the paternalistic old-school view of medicine. If patients have buy-in, they're more likely to be compliant. (A01)* |
|  |  |  | *I kind of have to go through their preferences with them, so there are some people that are very motivated and feel like eliminating things from their diet is something they could do. Those people will talk about different dietary approaches, like two-food, versus four-food or six-food, and what that means. […] Then if it's someone who really feels that's not something that they can do, then we talk about swallowed steroids as well. (A02)* |
|  |  |  | *I usually offer all of them, because I usually like to do kind of a shared decision making with the patient, because I feel like they'll buy into the treatment more if they get to make part of the decision. (A02)* |
|  |  |  | *I think that the shared decision making does help, and making sure they buy into the treatment options. It does help when they are very motivated to follow the treatment recommendations as well. I think that having a patient who is motivated and then buys into the treatment options, or the treatment that we choose—that really makes a good outcome. (A02)* |
|  |  |  | *…my philosophy it’s always a shared responsibility of trying of medicine, so explaining to the patient everything, try to educa[te] and teach as much about their disease and what is available out for the disease, and then together help make the decision as what's the best route for the patient to start with. […] The way I've always done medicine is it's really two-way; it's I'm giving them options, but I'm also trying to guide them into making the right decision for them as opposed to me making their decision*  *(A03)* |
|  |  |  | *Once I give all the information about the disease, about the different potential therapies, then I try to have them ask me as many questions about the different therapies out there so they can start working through what they're willing to do. Some of 'em, like my friend, you're not willing to even talk about the word needle, so it's not a good option for him, versus someone else is like, Yeah, I'm not pulling food outta my diet at all. I don't care what you say. Food avoidance is not gonna be an option. It's gonna be really directed by what options are they even open to and then you go from there. (A03)* |
|  |  |  | *…the conversation is a discussion as opposed to a lecture, so I'm drawing the parents or the patient in to see what they want, what do they want out of this, what are their goals? All my conversations [is] organic and directed by their questions and what they tell me they want. They're not very different. Yeah, they may end up in different spots, but how I get to there is very similar. (A03)* |
|  |  |  | *I’m just a big believer in autonomy, I guess. I don't live your life, and I don't walk in your shoes and if that's your choice, that's your choice. As long as I can inform you about what I think could help. I always try to give patients options ’cause I don’t want people to be like, Oh, there’s that option? I would have taken that if only I had known about it, right? I don’t know what fits in best with what you want for your life. I think I always try to just make sure that they know what treatments are available. (A04)* |
|  |  |  | *I try to find out why they wanna pursue that treatment first. Why is it you wanna do that and not—and what are your reasons? Because sometimes, you find out that the reasons are something that if you talk to them and explain that they realize aren’t valid reasons. (A07)* |
|  |  |  | *Maybe, I didn't take the time to assess how well they actually understand what I'm saying. Sometimes you just tell them, and you think you did your job, but do they actually understand or hear the importance of it? (G01)* |
|  |  |  | *Right off the bat, when they say no, I don't trust medications, so that's an easy one. You say, okay, well, we do have alternatives that we can work with. Food elimination, those are typically the one that we go to, talking about dairy being a really common allergen that they can work with. […] I have to listen to what the rationale is, what their particular preferences and if it's not reasonable then there might be some lack of understanding of your condition. (G01)* |
|  |  |  | *I’m not like, This is what we’re gonna do. I go, This is what the options are, what do you think? I’m not telling them what to do. I’m telling them what we can do and seeing what they think, and it’s like a mutual decision. I feel like that’s just the way that I practice medicine, so it’s not specific to EOE. (G02)* |
|  |  |  | *I think a lot has to do with patient’s interest, and what they think about the different options. I might have my biases about what they think that they’re gonna like. You know, diet. I guess if somebody was more into dietary changes, but nobody’s excited about restricting diet, you what I mean? I think that might play a part if somebody seemed organized enough and motivated enough about diet, but I really think nobody is excited unless they were gonna make those changes in their diet for other reasons. No one is excited about making those changes, but I guess that’s it. I feel like you get that, you get a hold of that information just by talking to them about what the things entail, and what they’re interested in. (G02)* |
|  |  |  | *I guess if they were more interested in one of the things that I had brought up in my descriptions of them, I would go into more detail if they would ask more questions, or if somebody was more interested in diet versus medications. That would certainly, I would be like, Oh, you’re interested in this? Let me tell you more. I think subjectively, it is based on their interest. I give them some basic information to start off with, and if they probe me for more information then I go, Okay, well I’ll give you more details. (G02)* |
|  |  |  | *I’ll get a sense of if they are averse to medications or vice versa, or they’re very wary of potential side effects. A lot of patients will volunteer that information. They’ll say, I really don’t want to be on medications, or I’ve heard PPIs do X, Y, and Z, or I’m worried about topical steroids and their potential side effects. Those are patients that may already be pretty averse to doing medical therapy. Those are patients that I’ll present to them dietary therapy. Some will be really into that. Other patients, again, it may just be challenging from a lifestyle perspective. If they have questions about PPIs or steroids or things like that, I’ll give them my usual spiel about those types of medications in terms of PPIs, in particular, they’ve been shown a number of studies that link them to certain other conditions. (G03)* |
|  |  |  | *Because in my mind, all three classes of treatment options are equally viable. I think it's more important to choose a treatment that's in line with the patient's preferences rather than being overly rigid. I don’t think there’s a lot of evidence to be overly rigid in that situation... (G03)* |
|  |  |  | *I’m not the patient. From a clinical perspective, I say, In general, that’s the goal is to, again, get your symptoms under control, prevent you needing to go in the ER or food impactions, things like that. In my mind, it’s not terribly the focus because it really should be about the patient and what is important to them. (G03)* |
|  |  |  | *If you provide them a choice, they're more likely to adhere than if I just tell them what they're going to do. (G04)* |
|  |  |  | *I usually go with the patient preference. If they verbalize what they want to do, I usually go with it, to be honest. The guy who wanted to do the diet, he told me. He’s like, I’d much rather do a diet. I didn’t try to convince him otherwise. I said, Okay. We’re gonna do the diet, let’s do it. If they give me a preference, I usually go with what their preference is. (G04)* |
|  |  |  | *I'm like, Look, it's your body, right? I'm not gonna sit here and tell you what to do. (G05)* |
|  |  |  | *I think that sometimes patients—you don't have their trust. Or you haven't established their trust, or when they're not responding, for instance, to the first medication that you put them on, and they lose trust in that relationship or don't want to continue for that reason. (G06)* |
|  |  |  | *The way the conversation goes is a lot of times patient driven. You see how they react to your cues. We tell them about PPI, and they are all big eyed, and start asking questions, explain why, ask why are you concerned to address that specific question. (G07)* |
|  |  |  | *I think it's important to just have a good conversation to understand why they don't wanna do that specific treatment. If they're not convinced, it's probably not gonna work. I try to incorporate what they want and make a joint decision. (G09)* |
|  | **Physician Information Use and Resource Needs** | Physicians used varied educational resources (e.g., pamphlets, websites, EMR smart phrases, diagrams) but identified unmet needs for standardized, comprehensive, patient-friendly materials and decision aids covering treatment options, risks, and current advancements. | *I like to draw in the exam room. I think that helps patients because they can visualize it. They can see. I draw the esophagus and the eosinophils and whatnot. I think that helps. (A01)* |
|  |  |  | *It would be really nice to have some sort of handout, or something describing all the different options and the risks and benefits. That would be nice, because I don't really think we have that right now. Yeah, I think something in Epic, where you can just click on, and it will add it to the patient instructions for them to print out, that would be nice. (A02)* |
|  |  |  | *I think it can be challenging to explain what EOE is too, so having a handout in patient language, explaining exactly what EOE is, and what the consequences of not treating it are, would also be good. You know, I can explain it in person, but then they leave and they forget. It would be good to have something to hand them as well. (A02)* |
|  |  |  | *I think the biggest thing is a better repository or a better website for the go-to […] a better website would be better, that's for the patients. […]Easier to navigate, because what they have right now is there is a lot of information spread all over the place about eosinophils,[…] but there's not a really comprehensive ‘this is EOE, this is everything about it’ where they could just go to that part of the website and know everything about eosinophilic esophagitis. (A03)* |
|  |  |  | *It would be nice to have like patient education handouts. That would be the biggest thing. For like a real blip in time, the American Foundation of Eosinophilic Disorders had pamphlets on EoE that they gave to us and that was really helpful because whenever you talk to someone, they retain like one percent of what you tell them in office, and it’s kind of a rapid-fire visit anyway, right? When you’re trying to really gauge how much do they really know, it’s so nice to have written materials to send them home with. (A04)* |
|  |  |  | *I think the biggest criteria for a patient education handout is that it should be not super verbose. It shouldn’t have a ton of words. It should be at the appropriate literacy standard for an American adult. It should not contain a ton of jargon, except for jargon that is necessary, so if you’re saying eosinophilic esophagitis, then you should be able to define eosinophilia and esophagitis in that. Those are the most important things, but I also think there should be a little blip about what is it and then what are symptoms and what are treatments. It should have a couple things at the bottom for them to have more information when they’re ready to search for it. (A04)* |
|  |  |  | *I guess there's a plethora of information, right? How do you distill it so it's easy for everyone, and you know that they got the material? I guess having handouts would be good. Like, a nice handout in the clinic of the major salient points. Maybe they have those at GI. I don't know. I don't have one, I don't think. (A05)* |
|  |  |  | *Figuring that sort of thing out like how do you feel about medications in general? How do you feel about—if we recommended avoiding foods, how realistic is that for you? That kind of information I think would be helpful. (A06)* |
|  |  |  | *Well, once I see the patient, I don’t let them just blindly go out, and I usually direct them to APFED, the American Partnership for Eosinophilic Disorders because it has a lot of really good information on it, and I tell them they can go to APFED. They can go to the practice parameters. I’ll give them links to that. One of the wonderful things about Epic as much as we hate it is we have these little dot phrases. I can just throw them in their after-visit summary, and I’ve got all the resources, say, If you wanna look, I think it’s important to look. You get more information. Here are good resources. Mayo Clinic has some good stuff, and I don’t let them just wander out there to get more information. (A07)* |
|  |  |  | *Well, resources they need we can’t give them is time. We have time we can spend with patients and a lot of the people. We’ll get patients referred to us from outside gastroenterologists, […] but we start telling them things that nobody ever sat down and talked to them about any of this, about EoE and what this is, and all these different aspects of it. Nobody ever said that ’cause they don’t have time. (A07)* |
|  |  |  | *The patient [information] would be basically what's EoE, what are the standard treatment so that everything we’re telling them, they have something to take home and read up on it instead of trying to dig through it in the internet. Something that's approved by [institution or clinic] that these are the information that's valid and correct. (G01)* |
|  |  |  | *I don’t know if there’s like a pamphlet that’s good for the patient, or something. […] I think it would maybe having some sort of pamphlet for the patients to read about the different options, and different resources that are available to them, and trusted resources that we trust that they should look into if they have more questions. (G02)* |
|  |  |  | *I would say where it would be helpful would be some sort of resource for patients with a new diagnosis that they can go to that can explain the condition and the treatment options ’cause, again, I think that period of time after a food impaction, you don’t have the time. The patient is still recovering from anesthesia. It’s usually in the middle of the night. It’s just not a good time to have a nuanced conversation like that. These also aren’t patients that I can see in the office the next day, necessarily. They have some sort of resource to either give or send patients to, in that setting, like when they’re discharged from the ER or something like that. Hey. Tomorrow go look at this website, or This’ll get sent to your house, or something like that. (G03)* |
|  |  |  | *This would be the right place to put a decision aid or something along those lines. If I had a go-to website […] or a piece of paper for which I say, 'This is your EoE, and here's your options. Which one does it sound the best to you, which I'm sure exists?' That would probably be helpful for patients, just in general. (G04)* |
|  |  |  | *I would like to see [it] be readable for the patients, be easy to understand, describe the medications available, the risk, and their side effects profile. Then what a diet would look like. I think those are the biggest things about decisions. I don’t think a decision needs to explain the importance of adhering. I think that’s my role. I think my role is probably to explain all of it, to be honest. To give them time to have something at home to think about, to look up, whether that’s on the website, or something, or a piece of paper, or an app, or whatever it is. (G04)* |
|  |  |  | *We have a eosinophilic esophagitis protocol here[…] It's this long, basically, flowchart. Typically, I'll actually give the patients the flowchart as well if we do go down that road because it really helps them to be able to follow along. They like to know that because if they're just going blind and they're like, Okay, it's confusing, and the next scope, they're like, I don't know why I'm getting this. Now they're eight scopes in, so it's much easier for them to have it. I'll typically give that to them as well. It's this long flowchart with different branches of, If this, then this, et cetera. (G05)* |
|  |  |  | *We have the handouts that we can give patients, but I think that it could probably further muddy the picture if they're getting all of this stuff about potential drugs and things. I think just having it available to us and then obviously making the patient instructions, obviously, as thorough as possible but more easily digestible is what I would suggest. […] The questions I'd always get are about, Okay, which foods? What data? What are the percentages? What are the other foods that can cause…?and so those kind of things are not in the actual handout. I think having something a little more detailed could probably help the patients. (G05)* |
|  |  |  | *In clinic, we just go to this small little file cabinet, pick out the handout on EoE, and hand—give them materials to read. Virtually, it's a little bit more challenging to do that. We can sometimes send it to them by mail. […]I think the pamphlet has just basic overview information which really, in large part, reinforces the information that I've told them in clinic or in the visit. I think patients digest information differently. I sometimes think about the fact that in medicine, you spend a lotta time explaining things in clinic. Sometimes what the patient actually recalls or remembers of that even though they look like they're listening to you, I think sometimes you as a provider don't realize that it's really not being synthesized. (G06)* |
|  |  |  | *I think providing a handout of some sort does help them because they're able to take that home and think about it a little bit more and really read it again. […] if they leave clinic, and they don't have any information at all, or they're looking on the Internet and things like that. Then sometimes the information gets clouded or distorted. (G06)* |
|  |  |  | *I created a [dot]phrase [in EMR]. That’s what eosinophilic esophagitis is. That’s why we explain. We send the treatments. I give them some links to the Up-To-Date, and some of the professional associations. (G07)* |
|  |  |  | *The SmartPhrase is something that we put in the patient’s result note that will go to their MyChart that will give a paragraph about eosinophils and allergies of the esophagus and outlining different potential treatments, including elimination diet, PPI therapy, steroid slurry, and usually suggesting an office follow-up. (G08)* |
| **PATIENT PERSPECTIVES** |  |  |  |
|  | **Information and Knowledge Seeking** |  |  |
|  | *Medication Preference Archetype* | Patients in this archetype tended to rely primarily on their physicians for EoE information, expressed skepticism about the quality of online sources, and preferred to verify information through their healthcare team. | *I don't actively research it, especially now that it's relatively under control. […] I joined that one group on Facebook, but to be honest, I don't like the opinions of people on the internet. A lot of it's repetitive. I think I'm following it but I don't get alerts or something. I don't know. I don't use Facebook that much. (P102)* |
|  |  |  | *There's so many fake news out there. (P104)* |
|  |  |  | *My mom sends me a lot of articles. Then whatever I’m told by my doctors. I haven’t done a ton of outside research… (P105)* |
|  |  |  | *In my limited opinion, there is not a ton of information out there about EoE in general. It’s still very new, and that makes sense that there isn’t a ton of research or insights into it. I guess this is a start (P105)* |
|  |  |  | *[I get information] from my physician and the notes that they share with me. I've done some online research, whether it's Google searches or whatever. I haven't read a lot about EOE specifically. […] Zero from social media 'cause I'm not on social media. […] WebMD comes up when I do these Google searches. Other than that, I would say just utilizing the resources that—I can't think of any other specific websites that I've looked at or pulled up—but utilizing the resources that the provider shares. (P106)* |
|  |  |  | *I feel like someone's circumstances can be different than my own, so it might not be as relevant. […] I just listen to what the doctor thinks should be done. […] Just whatever the doctor tells me, honestly. I could probably search things up online, but I've never tried to, so I don't know how accurate or helpful resources like that would be. (P108)* |
|  |  |  | *You could Google search something, but I don't know what would pop up that you could actually trust or not trust. (P108)* |
|  |  |  | *I just think there's a lot of this wellness push that's not rooted in medical information and just sends you further down the rabbit hole. I'm not gonna pursue different crafty things without input from the doctor in saying this is or isn't gonna be effective. I think it's silly to make treatment decisions without doctor's input. (P111)* |
|  |  |  | *There's a lot of wellness information out there that's not rooted in medical science that is a waste of time. I do think very critically about what I consume on the Internet and I cross reference it with my trusted doctors. […] WebMD can be a dangerous place, diagnose yourself with cancer every day. I think it's looking at things critically and cross-referencing it with my doctor. […] I love to see what kind of wacky stuff people put on the Internet. (P111)* |
|  |  |  | *I don't go to like Dr. Google because I know that that's not a good source of information. I like it from the medical professionals, the doctors, especially the GI doctors. (P113)* |
|  |  |  | *From the internet, no. I prefer to get it from a doctor, and more preferably, a specialist. If I look anything up online, I make sure to look at it with critical thinking skills and question data 'cause sometimes you get off-the-wall treatments [… ] I prefer a specialist or my primary care physician. (P115)* |
|  |  |  | *Never social media—I'm actually not on social media at all […] I guess reliable sources on the internet, just I guess treatments and symptoms. I haven't really looked at any of that kind of data in a long time, so I just trust my [gastroenterologist]. Yeah, that's who I would trust first and foremost. (P115)* |
|  |  |  | *It was just like we're trusting the doctor, because there's nowhere else to get this information. There's no studies being done at the time, or if there were, not publicly. There wasn't information about them yet, so it was just trust of the doctor to get the information. (P117)* |
|  |  |  | *I don't really go on social media very often to be honest with you. (P117)* |
|  |  |  | *I haven't read a ton about it, so I don't have any resources there either. (P118)* |
|  |  |  | *…anything that I read or see, until I take stock in it, I pretty much verify with my doctor. I don't want to go down the black hole of misinformation. (P119)* |
|  |  |  | *I like doctors, I like professional input. (P124)* |
|  |  |  | *I'd say you should talk to your doctor. I should probably do more research more often or I guess I do meet with my doctor often enough that she can tell me if there's anything new to be aware of. (P124)* |
|  |  |  | *I think when I get information online — medical information — it's usually something I use to bring up when I have an appointment with my doctor. It's not something I would make decisions about, typically. If it's something that is not invasive in anyway, then I might try something out that I read online, but I think that mostly I'm doing research so that I'm prepared to know how to talk about it with my doctor. (P124)* |
|  |  |  | *Primarily, I get my information from my doctor, my primary, or the specialist if I’m seeing one. I do go simply research or Google or WebMD, or whatever, just general information out there that you an get. You never know when something new might pop up. I do look online at times. I primarily work with my doctors. (P125)* |
|  |  |  | *I get 99 percent of it from the Michigan doctors. (P131)* |
|  |  |  | *I think everything has come from the consultation with the doctor. […] I don't look at social media stuff related to it or groups or anything. (P133)* |
|  | *Natural Treatment Archetype* | Patients in this archetype tended to be the most proactively knowledge seeking, frequently researching EoE online from credible sources and emphasizing the importance of self-advocacy in healthcare. | *I did a lot of research on the computer -- a lot of YouTube videos. I listened to a lot of professors speak at universities, and I tried to go with credible sources. (P110)* |
|  |  |  | *I’ve read a lot of the studies. I do read studies. I listen to physicians, I listen to scientists, and I know that there’s lots of different studies out there, but as long as I—my doctor and I finally agreed that as long as I could keep my numbers where they were, then we wouldn’t discuss it anymore. (P110)* |
|  |  |  | *…* *the doctor, so she had discussed the condition with me and offered me treatment options, but I was also really well educated on the topic, so I sort of knew that there was one very obvious treatment option for me personally. (P112)* |
|  |  |  | *For me, 'cause I'm very knowledgeable in health, nutrition, and medicine, I read journal articles, and then I talk to the doctor, but I'll occasionally see what's the newest research on the field… (P112)* |
|  |  |  | *Most of it I've researched myself online, and just gone to the different hospital websites and the different people who are researching it. I've joined some Facebook groups. I don't always rely on that, just because there's so many people with so many different experiences than me that I'm like, "Ooh, that's not my EoE experience," but I get to see what their treatments are. Then I go sometimes say, "Oh, well, what's that medication? What are they taking?" Then I'll go research it. I'm just a research guru, so I get on and just read up on everything. (P120)* |
|  |  |  | *I had a routine blood test done for my annual physical. It had elevated eosinophils. It's like, "Oh, I'm curious. I don't know what those are." I went and I googled it. I'm reading it, and the next article down was EoE. I didn't click on it. I went to bed that night and my brain went, "Why didn't you read that?"*  *I got up the next morning, googled the same thing, read that article, printed it out, and just gave it to my husband. He's like, "That's what you've been telling me. That's your symptoms." I called U of M and got an appointment. (P127)* |
|  |  |  | *Ask all the questions. I feel like no question is too dumb. But really ask all the questions. Definitely get into the EoE community. I’m on this on Reddit and it’s been very helpful just hearing other people’s options, what treatments they’ve done. What works well, what hasn’t. Not that it would be the same for me, but it’s interesting to just get people’s questions and I’ve definitely asked questions on there. Ask questions and make sure that you have a provider that’s gonna answer those questions. If you don’t, just be pushy about it. I hate that we have to be all our own advocates, but you need to be your own advocate in your own healthcare journey. Be pushy. Ask questions and make sure that you have a provider that's going to answer those questions. If you don't, just be pushy about it. I hate that we have to be all our own advocates, but you need to be your own advocate in your own healthcare journey. Be pushy. Ask the questions. (P132)* |
|  | *Treatment Ambivalent Archetype* | Patients in this archetype tended to be less proactive in seeking EoE information, tended to rely on their physician or social media sources, and were less likely to seek out formal or evidence-based resources independently. | *I've gotten it all [my information] through my doctor at U of M. (P103)* |
|  |  |  | *Just my doctor, but I'm trying to do my own research. Isn't there something else I can do? I'm starting to read you have to eliminate foods like maybe dairy and other foods that are triggering it. (P109)* |
|  |  |  | *I go to YouTube and hear other people's stories or I go to the discussion groups on Facebook…. (P109)* |
|  |  |  | *There's communities online of people who have EoE that you can learn. On Facebook, on Reddit, there's all these places that there's just big groups of people who have gathered together who have EoE. It's interesting—like I said before—to see those similarities between everybody. Everybody's got these tricks too. (P122)* |
|  |  |  | *Whether it's like Google Scholar, I'll look at that sometimes, or Reddit, or Facebook. People on the Facebook groups post the studies so you can see 'em. Everybody who has EoE and who is in one of those Facebook groups or Reddit groups is super informed about it, because everybody's constantly sharing the newest developments and the newest drug trials. (P122)* |
|  |  |  | *{My wife] did most of the internet research and talked to the doctors as much as I did. (P128)* |
|  |  |  | *[I get my information] from the doctor. I just go on Google for something. (P130)* |
|  |  |  | *One of my doctors had sent me some resources. I'm sure the link is in my portal profile, but I haven't really looked into anything EoE related for a while. (P130)* |
|  | **Decision Making and Communication** |  |  |
|  | *Medication Preference Archetype* | Patients in this archetype tended to prefer physician-led decision making, placing high trust in physician expertise and preferring to follow medical guidance, while still valuing clear communication and explanation from their physicians. | *[Why doctor should lead] They are the ones that have the experience, assuming it's a good doctor. (P102)* |
|  |  |  | *I like to listen to my doctor. (P104)* |
|  |  |  | *I just did what I was told [by doctor]. That was really it. Sometimes I didn’t, and it didn’t work out for me. Well, I guess it sometimes worked out for me. Yeah, just do what you’re told, I guess. (P105)* |
|  |  |  | *I was really just leaning on what the doctor said. I agreed to that, just trusting the knowledge and the recommendations of the provider. (P106)* |
|  |  |  | *I would say listen to your doctor. I think we all could be better patients at times. I certainly can. Follow those instructions as diligently and consistently as possible. Don't take that for granted. Try to get into a routine where you can be mindful and, I think, disciplined, again, with respect to your care instructions. (P106)* |
|  |  |  | *…the doctors know what they're doing, so I need to make sure that I'm following it exactly so I can improve as much as possible. (P108)* |
|  |  |  | *The doctor is educated on this, and I am not, so I'll just follow their opinions. I'm not a doctor, so I should not be making medical decisions without consulting one. […] I just listen to what the doctor thinks should be done. (P108)* |
|  |  |  | *I am not a doctor […] I'm gonna stay in my lane. (P111)* |
|  |  |  | *I think it's silly to make treatment decisions without doctor's input. Like I said with my PCP, when I talked to my PCP about not doing anything for a period of time, he was like, "How's that gonna work out for you? Let's talk about the long-term effects." That was an important discussion. (P111)* |
|  |  |  | *It's not helpful for a doctor to be like, I'm a doctor and I know everything so just do this. Explain to me what's going on inside my body that this is the treatment you think is appropriate for me. (P111)* |
|  |  |  | *I think that I chose to go this [treatment] route because my doctor was pretty reassuring about the safety of omeprazole and I trust their judgement. (P124)* |
|  |  |  | *I like doctors, I like professional input. (P124)* |
|  |  |  | *I think the first time I had the EGD, I never heard any real follow up from the doctor, and that was not a good thing. I should have been much more proactive in trying to understand what the results were and what that meant for long term or what follow up I should have, and I think that that didn't happen after the first doctor that I was with. It happens frequently where you get a report and you're like, I don't know what this means and they're not calling me to tell me anything's bad, so if I feel generally okay — but I think I finally understand what works for me and what the recommendations have been from my doctor, and what the implications are of following or not following the treatment plan. (P124)* |
|  |  |  | *I’ve always trusted the physicians that I deal with in this area and felt comfortable with them. I would like them to take the lead. […] I trust my doctors and work with them when I do have problems. (P125)* |
|  |  |  | *I don't make any medical decisions without running through my doctor. […] I like my doctor's input. I would never do anything without getting their input. (P125)* |
|  |  |  | *I appreciate the doctor's opinions and that's why I go to see them. (P126)* |
|  |  |  | *I went with [the treatment] 'cause obviously I trust [the doctor’s] opinion. (P126)* |
|  |  |  | *I do not like to make my own decisions. I like to talk to the doctor. (P131)* |
|  |  |  | *I'm not a doctor, so I'm gonna rely a lot on what the doctor says (P133)* |
|  |  |  | *It's something medical-related. I have no expertise in that area, so I would heavily rely on a doctor to give me some direction and give me options and give me advice. (P133)* |
|  | *Natural Treatment Archetype* | Patients in this archetype tended to prefer a balanced, collaborative approach to decision making, valuing open dialogue with knowledgeable physicians. | *I actually like to make a lot of decisions about my EoE on my own, but I do take input from the doctor. I do advocate for myself. (P110)* |
|  |  |  | *[Do] you wanna follow your doctor’s advice, or do you wanna have your own opinion? I will say, I wanted [it] to be a marriage of input (P110)* |
|  |  |  | *I felt like [the doctor] really listened to me, but she also had some good ideas. We kind of came together with a plan. (P110)* |
|  |  |  | *I guess the doctor, so she had discussed the condition with me and offered me treatment options, but I was also really well educated on the topic, so I sort of knew that there was one very obvious treatment option for me personally that it really wasn't a very hard decision to make. […] so I guess the getting the doctor's input was important, but I knew so much about the condition and what I was gonna do a priori that it was more about what I needed to do rather than what I chose to do. (P112)* |
|  |  |  | *I know how I feel and what certain things, how they make me feel better than maybe the doctor would." (P116)* |
|  |  |  | *…listen to your body and try to see what you can glean on your own. Doctors are very helpful but they're not there on the day-to-day… (P116)* |
|  |  |  | *Just knowing your options and having a doctor that is open to what you wanna do, not pushing one thing or another. I do remember when I was like, I'm not gonna give up dairy and wheat and gluten and soy, it's just too much. There was a little bit of pushback from my doctor. Just having someone that's considerate and open to what you wanna do, even if it may not be the most rational decision. (P116)* |
|  |  |  | *All through the conversation on the phone, I felt like [the doctor] was not that informed about it, and very timid. He was very, "We could try to do more dilation, but I'm really concerned about a tear that would be a medical emergency." He didn't give me a whole lot of confidence in him. That's why I started looking for other doctors […] all-in-all did not seem like he felt equipped to really handle it, so I took things into my own hands. I tried the medication. Didn't really like it. I don't know. It didn't do anything, didn't seem to help. That's why I started looking for a second opinion." (P120)* |
|  |  |  | *[It] was the only time I've ever had a doctor sit and talk to me that long. I felt good because I had researched it all. What she told me, none of it was a surprise. To hear her sit down and talk to me, and talk it through, and then also validate that you're a person, this is your life, this is your choice, so you can go back and you can choose this treatment right now, and you can choose this one later, and then you can come back to this one, and you can go back and forth. You can do whatever you wanna do at any time. Whatever makes life better for you. To me, that was like, wow, you couldn't have made it any better or easier than what you did, because I had options. There was no shame and there was no pressure, nothing. (P120)* |
|  |  |  | *I like when doctors communicate with me and talk through me like I'm a person. […] I just want it to be more of an open dialogue instead of one way… (P132)* |
|  |  |  | *I feel like I’ve had an experience like that with my EoE and I really disliked it. […] It just felt like someone wasn’t talking to me as a person with EoE. I was just told what to do. […] I find that very irritating and very frustrating. (P132)* |
|  |  |  | *I do like to have my input, but I also want doctor’s input. (P132)* |
|  | *Treatment Ambivalent Archetype* | Patients in this archetype tended to prefer a shared decision-making approach in which physicians present information and options, with patients having the final decision rather than simply deferring to physician recommendations. | *… they didn't give me any options. They were just like, "Here. Just take Flovent. We'll check back on you in a year," and it got worse. I noticed my symptoms are a lot worse, so I asked for the endoscopy 'cause something doesn't feel right. I got even more worse. (P109)* |
|  |  |  | *It just sucks because I haven't even been face to face with the G.I. doctor and everything's been done by the nurse just sending me a message through a portal. I wish I could actually sit down with the G.I. doctor, get educated on this, and different option plans than [them] saying, "Hey, just take this and we'll see you in a year." (P109)* |
|  |  |  | *They didn't even explain what EoE was. I remember the first time they diagnosed it with me and they just said "It's curable. You just need to take this and your symptoms will get better.” (P109)* |
|  |  |  | *I think that some of the previous gastroenterologists that I've talked to, they tell you take a PPI. Then you come back and they're like, "All right, we can try steroids. That's not quite enough in my opinion.*  *[…] I would rather that we have a conversation with doctors. The doctors tell me about the research, tell me about what works right now for most people. Then we make a decision together. (P122)* |
|  |  |  | *I think it has to be a discussion, because EoE it's just such a weird thing individualized for everybody. You gotta really, really understand what that particular person is going through. (P122)* |
|  |  |  | *Ultimately, you've gotta be making your own decisions most of the time, but it always has to be that, that discussion. If something feels bad to you, don't do it. If it feels bad to eat fruit when you're having a flare up, don't do it. If it feels good to drink something caffeinated when you're having an impaction or after an impaction to make you feel better, do that. (P122)* |
|  |  |  | *[Previous docor’s approach] was not a democratic form of medicine. She told you what to do, but she did not believe in steroids. […] She said, "Every couple years we'll go in and stretch it." That's what I did the first five or six years. Every couple of years, went in and got stretched." (P128)* |
|  |  |  | *most of the time, these decisions are gray not black and white. The days of not asking the doctor what's going on, just taking their advice is over. (P128)* |
|  |  |  | *I think it's important for anybody when they're talking to a doctor to not just follow their lead blindly, to actually question them. When they can’t answer your questions, it’s good that you asked them. Why would they care more about my health than me? (P130)* |
|  | **Patient Resource Needs** | Regardless of archetype, patients wanted educational and decision-making resources that were accessible, clear, trustworthy, and tailored to their individual circumstances, with some also expressing a desire for peer connection and support. | *I think if there was a timeline like, here's day one—if we were to do this food elimination diet, this is the amount of time it's gonna take. Not saying it's gonna work. Say we brought in a trigger food instead of—ended up not being a knot one so then we have to reverse that, about how long that study's gonna be versus the medication study. Then okay, "Well, this is what you're gonna do for the rest of your life. We got it under control. We got your medication dialed down. You're gonna have scopes every so often." I don't think I've ever seen a timeline like that. (P102)* |
|  |  |  | *I was provided with a list of some foods. It'd be nice if there was an app that you could scan, I know there probably is now, that would show you like, okay, this is food I shouldn't eat based on allergies… (P103)* |
|  |  |  | *I think like a paper or something about the different kinds of treatment options, including things that are being worked on, that might be new, and new research on it would be at least interesting to me." (P103)* |
|  |  |  | *My first thought was, “Yeah, maybe some statistics on how often this works,” […] I feel like it’s so specific to the individual. There’s a template that you follow, but then everybody fills it in differently… (P105)* |
|  |  |  | *I think leaning on the experiences of others is probably something that would be helpful and something that I haven't asked for and certainly haven't intentionally sought out. I think that would be helpful—someone sharing, "This is what I went through. This is how I managed. This was successful for me." I think that would've been helpful. I would love to know if there's something specific from a dietary perspective that would be helpful for me. To my knowledge, we haven't really identified anything that might amplify or exacerbate my condition or might help. I think that would be something that would be helpful too. […] maybe a focus group or folks who put their names in some sort of database, I don't know—something like that. It certainly could be just an online resource where folks just share their stories or share their best practices. That's something that we can access through some sort of portal. That would be fine too. (P106)* |
|  |  |  | *I think utilizing maybe the patient portal for those types of patient experiences would be fantastic. I know we have a office of patient experience. I don't know to what degree it's outward facing for patients to access that for other patient stories or other best practices. I do think that we could probably leverage our online internet resources to share some of those and leverage, again, patient experiences for those who are in this kind of care, treatment space. (P106)* |
|  |  |  | *More accessible information from verifiable sources. You could Google search something, but I don't know what would pop up that you could actually trust or not trust. Maybe, for example, a university sponsored or university verified information source that can give you actual tips on how to treat it aside from what they're being given, or just facts and information about things regarding the disease, certain potential environmental causes of it 'cause maybe it could be allergy induced as well, or maybe certain foods. Small things like that could be extremely helpful, I think, so just information sources like that. (P108)* |
|  |  |  | *I want it to say what is EoE and from there, maybe references where you can go and learn more about it. (P109)* |
|  |  |  | *…usually you hear they're gonna give you handouts if you're gonna take your vaccinations so you can read about it. I didn't get a handout. I didn't get anything. It was just like, "You have EoE.” "Okay, what is that?” (P109)* |
|  |  |  | *Support groups, even. Sometimes it's really hard to manage it and you feel like you're dying (P109)* |
|  |  |  | *For me, it's more helpful if people explain the why and how or science behind something […] a really simple explanation of things. Keep it simple, it doesn't need to have all the liability disclaimers on it. Keep it simple and give me a visual and fewer words to be like, oh, yeah, that makes sense. Or if you want more information here's a reliable link. I think in the patient portal, giving people reliable links to if you want more information or more understanding so that people don't go out to the World Wide Web and self-diagnose and use whatever unreliable sites they want. (P111)* |
|  |  |  | *Pamphlets are nice. If I'm going to a specialist, having the TV on those sites that actually talk about the ailment. Pamphlets with information, or pamphlets that guide you to certain websites, is something that I would like. (114)* |
|  |  |  | *I like in writing, or if there is a link to a website, or videos, I enjoy that with diagrams and things like that. Diagrams of the esophagus so you can get an understanding of what's happening physiologically 'cause I like to know things and understand things, so any of those methods. (P115)* |
|  |  |  | *It's helpful just to be connected to people that are going through a similar thing and have similar struggles. Like I said, everybody has a different experience so there's lots of different opinions, but it's good to just stay connected in the community in case there's some new treatment or someone found something that's really helpful or a resource guide. it's good to just have it out there in case you're looking for something or you might find something that's super helpful. (P116)* |
|  |  |  | *…* *if I was diagnosed now, I'd maybe wanna see some research or hear from other people. Just thinking about maybe side effects of the medication. (P116)* |
|  |  |  | *Maybe a verbal summary of the high points and then back it up with here's the actual study or here’s some written explanation, but having just an oral summary of, this is the gist of it, and then here's more information if you wanna go into the details. (P116)* |
|  |  |  | *Even just something as simple as a pamphlet explaining what EOE is would've been helpful[…] Just like something to say, hey, EOE is causing this to happen in your body. I didn't even know what it stood for a couple years. I didn't. […] Like a one page. Just something simple like that, that just goes through what's happening, what can potentially be causing it, and that sort of thing. Just to give you some sense of knowledge of what's going on so you're not lost. (P117)* |
|  |  |  | *…* *maybe knowing more about what other people had done and what their experiences were doing those things. […] I guess honestly, it just goes back to maybe patient testimonies. That would be important for me. (P118)* |
|  |  |  | *… if I have to follow something, but also has clear cut goals, has a clear-cut process. [...] A timeline would be really helpful. […] what do we do going forward? What does this look like? I realize that every case is different, there’s uncharted territory. I completely understand that. More of a like “hey, here’s what you can maybe expect, here’s a point where you might want to call your doctor, here’s a point where you might want to try this.” […] is this something that I’m gonna wake up tomorrow and feel fine again or is this something that’s gonna last? […] Almost like a way of saying hey, if this happens you need to call us. If this happens, maybe wait a day or two until you call us ’cause I think a lot of times I probably end up waiting too long to call. (P119)* |
|  |  |  | *Honestly, talking to somebody else who had it would've probably helped tremendously. Even if they'd only taken one avenue, I think that would help a lot. […] Just talking through it with somebody who’s been through it, knows a few of the pros and cons outside of the medical boundaries, I think would’ve been very helpful. Even if I didn’t even take their advice, just hearing another person’s perspective.*  *(P119)* |
|  |  |  | *If there had been a concise handout that had these are the treatment options, what were they, how they worked, what their success rate was, and here's where you can read more kind of a thing—having it concise would definitely be good […] To have something listed—I almost think in levels of ease, like start with the elimination diet, then do this, and then try—whatever, up to the most extreme, you have to be eligible for this medication. Have that all in one spot would be great because then I would know that, if this one stops working, what is the next level that I could go to. Yeah, that would be super helpful. (P120)* |
|  |  |  | *Maybe something on finding support groups. There was one about reading other people's experiences or hearing about them or whatever, but maybe a little bit more on support groups and things like that, because that is one other great piece of information to have, and to talk to other people so you don't feel like you're the only crazy one who can't swallow. (P120)* |
|  |  |  | *The more that I started finding people's stories and their symptoms that went along with it, I went, okay. This makes me feel not as much of a medical mystery. That was nice to hear. (P121)* |
|  |  |  | *I would like to see every symptom that could be related to EOE, or maybe the most common. […] I would also like to see maybe support group information on there. I would like to see treatment plans that go both ways, like traditional and alternative medicine. […] if there are specific doctors that specialize or that are recommended… (P121)* |
|  |  |  | *Then joining the communities, on Reddit, on Facebook, wherever you can find. Those are the two places that I found big groups of people who have EoE who talk about it. Join those and just read. It makes you feel better to read about other people who are commiserating almost in some cases. It's therapeutic to commiserate with people. Or, to share success stories, or to share tactics or to share things that work. (P122)* |
|  |  |  | *[Helpful to have] a really good description of the elimination diet and what the goals of the elimination diet are. Why are we doing this? What are the six groups? Then tell me what are the most common ones. [...]Then all of the medication options, PPIs, what do they do, what are the downsides? Steroids, what do they do, what are the downsides? Then the biologics, what do they do? What are the downsides? All listed on a sheet, a one pager, so you can quickly evaluate. (P122)* |
|  |  |  | *I've used those pamphlets that the doctors give. Probably, again, symptom management and what are the treatments and what have you. (P123)* |
|  |  |  | *If I could have some sort of booklet that has all the information about it and that has information on what the process is for different treatment options. Sometimes it's hard for me to take in all the information that I'm given in a conversation. I'm much more someone who likes to read through information and then be able to compare it without just having that as a part of a one-time conversation. If there was materials that I was given that had more detailed information that was not just scientifically based, because I think that's pretty useless to me [if] I don't understand it. (P124)* |
|  |  |  | *I'd say just what is it? What’s the research about it? What are the different treatment options? What are people's experiences with it? And maybe directing you to websites where you can continue to get updated information on it. Maybe communication through U of M's portal. I guess I just wanna know more about, I think, everything about it. Feeling like I'm up to date and that if there are other tests that come up or other treatments that come up that there's a way that I'm made aware of that without having to do my own research. (P124)* |
|  |  |  | *EoE, it’s not very common. I guess an explanation of what it is, not too in-depth, but enough that you have a good understanding of it. Treatment options and also the ways of possibly managing it, so that it gives you information about elimination diets or if it’s severe, what dilation involves. For me, I’m always interested in experimental or new technologies or medications that may help the situation, whatever it is, not just EoE, but anything. I would like to see information that may have something like clinical trials or something along those lines… (P125)* |
|  |  |  | *I think pamphlets are interesting, but I prefer here's a link to what we have, here's another link to what we could try. That way, if I had questions in the moment, I might find them out via those links. […] Obviously, maybe a less clinical description of EoE that is more layman's terms. I don't mind reading that stuff and I can make assumptions, but if it's easier to understand and if it's said in a way that, "Okay, this is what's happened to your body and maybe it is because of this," and then the various treatments options to you and just basically what those are all about, and just options for lifestyle management or someone to talk to in the moment, if there's a chat option. Those things are interesting. I know that's probably a little more difficult to pull off, but anything that gives you some direction on how to proceed. Like, what do I do next? What's the process, A to B to C? I know things may change or you may be able to may be up, may be down, you have to maybe try this and it doesn't work and you go over here, that kinda thing, but it doesn't have to be all of it, but at least here's what you think you have, here's some options for you to treat it, here's what you might experience via other people's experiences. Those are important. (P126)* |
|  |  |  | *It was just pamphlets and paperwork about what EoE is. I think I was directed to a couple of different websites. Again, those are geared more towards children. It's still very useful information. Yeah, there were a couple website links in there that I went and checked out. Then, they directed me to a Pinterest board […] when you're eliminating everything, it's hard to find vitamins that don't have some of the products in it, especially with soy. That Pinterest board, I was able to look for different things to fill my needs of either vitamins or protein bars or something. That was helpful. I found several products, some that I could find locally and use in my food preparation. (P127)* |
|  |  |  | *EoE treatment information in clear language--all the options and why, pros and cons of each one, and why this one is being recommended […] the doctor discussing my case and my treatment options and my risks. That's helpful, but the pamphlet 'What's EoE?' that doesn't help so much 'cause I can just—these days just get on one of the websites and pull off "What's EOE?" and you get the same information. (P128)* |
|  |  |  | *I’ve never met anybody else that’s had the EOE diagnosis. Then I wish I knew more about the potential side effects of EOE and long term what it means for me. I wish I had just gotten a little bit more information on that. […] Even having a handout, like written information, is helpful for me ’cause I do forget. […] Just a brief overview of treatment options would be nice. This is all in a handout, so exactly what it is, what potential causes, things that could make it better, both pharmacological and nonpharmacological, and risks and benefits of treating it versus not treating it. (P129)* |
|  |  |  | *I would like to know what the percentage of people that have EoE, what causes it, I think. […] I don’t know if there’s other reasons besides food, but I think that would be what I would like to see in the brochure. (P131)* |
|  |  |  | *If you could make something that basically had all the options and talked about if your priorities are blah and blah, maybe this will be the one for you. If you wanna just immediately feel like you want it to be the quicker fix this might be the option for you […] if you could make a fact sheet or a flyer or just a packet or something that had all that in there, that might have been nice […] because then you have a resource you could flip through. Maybe it has links to resources that you can investigate. You could link to Reddit, and you know it’s all there for them to get that information. […] I feel like would have been nice just to have a resource that then you could immediately, like, “Oh. We think you have EoE. Here’s a nice little information packet”. […] Probably like, what is EoE, a really succinct clear description of it […] Probably the treatment options link. Not the pros/cons, but like, if you choose this one option, this is what you can expect for each of the treatment options. Very brief. Doesn’t have to go on—if it’s only a one pager, but maybe something that just gives a brief overview on what to expect for each one. I mean if you can do additional resources, probably here’s links to resources if you need more information, or you wanna investigate it more or you have more questions. Something like that would have been nice too, I think. (P132)* |
|  |  |  | *I think the doctor did a pretty good job explaining everything and what it is and what it looks like and what the options are. Plus, having the pictures of the scopes was helpful, but other than that, if someone said, "You have EoE," and not knowing what it was or anything, if there's some sort of literature, pamphlet, or something with pictures and stuff, that would help. […] a visual of what it is and what it looks like inside your esophagus and then what the treatment options are and what food groups are normally eliminated to try to fix it that way or what the medication options are. Again, kind of like how it was laid out to me by the doctor, just in a written form. (P133)* |
|  |  |  | *What is it? What causes it? Then, what are a few examples of it? Maybe some pictures or diagrams ’cause everyone loves pictures and diagrams. (P134)* |
